# Asymmetry in cortical thickness and subcortical volume in treatment-naïve major depressive disorder

**DOI:** 10.1016/j.nicl.2018.101614

**Published:** 2018-11-28

**Authors:** Zhiwei Zuo, Shuhua Ran, Yao Wang, Chang Li, Qi Han, Qianying Tang, Wei Qu, Haitao Li

**Affiliations:** a Department of Radiology, Affiliated Southwest Hospital, Army Medical University, #29 Gaotanyan Main Street, Chongqing 400038, China; b Department of Psychology, Affiliated Southwest Hospital, Army Medical University, Chongqing, China

**Keywords:** Major depressive disorder, MRI, Cortical thickness, Subcortical volume, Asymmetry, AI, asymmetry index, DLPFC, dorsolateral prefrontal cortex, GLM, general linear model, HAM-D_24_, 24-item Hamilton Depression Scale, MDD, major depressive disorder, SAS, Self-rating Anxiety Scale, SDS, Self-rating Depression Scale, VBM, voxel-based morphometry

## Abstract

**Background:**

Numerous cognitive and emotional functions are executed asymmetrically between the left and right hemispheres. Right hemisphere hyperactivity/left hemisphere hypoactivity often appears to be a feature in neuroimaging studies of depression. However, few studies have evaluated abnormalities in structural asymmetry in untreated patients with major depressive disorder (MDD).

**Methods:**

In this study, 3-dimensional high-resolution structural magnetic resonance images were acquired from 35 treatment-naïve patients with MDD (mean age = 28.9 years, 22 females) and 35 normal controls. The asymmetry index in cortical thickness and subcortical volume were calculated based on an automated surface-based technique.

**Results:**

Abnormalities in structural asymmetry in patients with MDD were mainly located in the cortical-striatal-pallidal-thalamic circuit, including the superior frontal cortex, rostral middle frontal cortex, caudal middle frontal cortex, nucleus accumbens, pallidum and thalamus. No significant correlation was observed between symptom severity and asymmetric measurements.

**Conclusion:**

These findings provide further evidence for the altered morphological interhemispheric imbalances in depression and these alterations were independent of depressive symptom severity, suggesting that cerebral asymmetry could be an appropriate indicator of morphological variations in mental disease.

## Highlights

• Cortical asymmetric changes in depression mainly involved the frontal cortex.
• Clinical variables were not associated with cerebral asymmetry.
• Cerebral asymmetry could be an indicator of morphological variations in depression.

## 1 Introduction

Major depressive disorder (MDD) is a psychiatric disease that seriously impairs patients' physical and mental health. MDD is characterized by a “negative cognitive triad”: a persistent negative idiosyncratic appraisal of the self (worthlessness), the future (hopelessness) and the world (helplessness). Major depressive episodes may cause a person to feel that life is not worth living and to commit suicide. Depression is the leading cause of disability and poor health worldwide (Lopez and Murray, 1998; Ferrari et al., 2013); therefore, understanding the pathogenesis and pathophysiological processes of this disorder is increasingly important.

Although many neuroimaging and autopsy analyses have revealed brain abnormalities in patients with MDD (Andreescu et al., 2008; Zhang et al., 2016; Bora et al., 2012; Pizzagalli, 2011; Reynolds et al., 2014; Schaffer et al., 1983; Grimm et al., 2008; Fonseka et al., 2016), the precise cause and mechanism of this condition remain poorly understood. Volumetric changes in gray matter in patients with MDD have been researched extensively in previous anatomical studies (Andreescu et al., 2008; Zhang et al., 2016; Bora et al., 2012) and are thought to be located in cortical-limbic areas including the anterior cingulate cortex, dorsolateral prefrontal cortex (DLPFC), orbitofrontal cortex, amygdala, thalamus and putamen (Andreescu et al., 2008; Pizzagalli, 2011; Bora et al., 2012). Abnormalities in these regions impair various cognitive and emotional functions, such as attention, performance monitoring, self-evaluation, and behavioral adjustment (Pizzagalli, 2011; Reynolds et al., 2014). Many cognitive and emotional functions are executed asymmetrically between the left and right hemispheres. The valence hypothesis (Wittling and Roschmann, 1993; Davidson and Irwin, 1999) has been proposed to describe the relationships between emotion and cerebral asymmetry, alleging that negative emotions are associated with the right hemisphere and that positive emotions are associated with the left hemisphere. MDD is a kind of affective disorder, with a rightward activated pattern of frontal alpha asymmetry often appearing as a characteristic in electroencephalography (EEG) (Chang et al., 2012; Schaffer et al., 1983; Zotev et al., 2016). Frontal asymmetry in the EEG alpha band signal is thought to correlate with withdrawal behaviors and reduced approach motivation. Functional MRI (fMRI), perfusion MRI and PET studies have attempted to identify specific brain regions showing these functional interhemispheric differences in depression (Grimm et al., 2008; Phillips et al., 2003; Chen et al., 2016). However, the structural asymmetry in depression has not been thoroughly investigated.

Structural asymmetry is used to evaluate anatomical differences between the left and right hemispheres by eliminating the impact of brain size; it is determined early during human development and seems to be related to asymmetric gene expression between the hemispheres (Sun et al., 2005). One recent report investigated cortical volume asymmetry in the DLPFC in MDD using the region-of-interest (ROI) approach with voxel-based morphometry (VBM), and found decreased asymmetry in medication-naïve patients with first-episode MDD (Liu et al., 2016). Nevertheless, as one of the most common methods used to measure changes in cortical volume, VBM may underestimate the subtle cortical differences because of heavy imaging smoothing and substantial cortical folding (Jarnum et al., 2011). Instead, another commonly used cortical measurement, namely, cortical thickness, is more sensitive to disease states (Pereira et al., 2012; Asllani et al., 2016; Meyer et al., 2014). In the context of MDD, cortical thickness has been suggested as a candidate endophenotype (Peterson and Weissman, 2011). To the best of our knowledge, asymmetric alterations in cortical thickness and subcortical volume in patients with MDD have not yet been reported. A comprehensive understanding of the cerebral pathophysiology changes in depression is essential and may lead to more targeted approaches for the prevention and treatment of MDD.

In the current study, whole-brain analysis methods were used to systematically evaluate asymmetric abnormalities rather than making a priori assumptions of the locations. We recruited currently depressed patients to rule out the neuroprotective effects of pharmacotherapy and enable a direct assessment of underlying state-related changes in patients with MDD. Based on the existing literature (Liu et al., 2016; Kumar et al., 2000; Grimm et al., 2008; Phillips et al., 2003), which found right-lateralized volumetric/functional asymmetry in patients with MDD, we postulated that patients with MDD would show right-lateralized cortical thickness changes in some brain regions, especially the frontal areas. We employed a surface-based approach to examine these specific parameters and test these hypotheses in untreated patients with MDD.

## 2 Materials and methods

### 2.1 Subjects

Forty medication-naïve, patients diagnosed with MDD were recruited as potential participants from the outpatient clinic in the Department of Psychology of Southwest Hospital, Chongqing, China. Depression duration was assessed in an interview using the life-chart methodology. The inclusion criteria for patients were: (1) age 16–48 years; (2) meeting the Diagnostic and Statistical Manual of Mental Disorders IV (DSM-IV) diagnostic criteria for MDD; (3) currently depressed but not receiving any drug treatments; and (4) a total 24-item Hamilton Depression Scale (HAM-D_24_) score > 20 (moderate severity). The exclusion criteria included a history of bipolar disorder, schizophrenia, schizoaffective disorder, psychosis, bulimia, seizures, obsessive-compulsive disorder, primary posttraumatic disorder, anorexia, alcohol abuse, substance dependence, suicidal behavior, or brain injury or any contraindications for MRI. Thirty-five patients (22 females) met these criteria and were included in the study.

We also recruited 35 age-, gender- and education-matched normal controls (NC) who had no history of alcoholism, drug dependence, psychiatric disease, traumatic brain injury, chronic medical disease (such as heart failure), or any neurological diseases.

All participants were right-handed. Independent neuropsychological evaluations including the HAM-D_24_, the Self-Rating Depression Scale (SDS), and the Self-Rating Anxiety Scale (SAS) were administered to all participants by two experienced psychologists. All participants included in the study provided written informed consent. The study was approved by the Medical Ethics Committee of the Southwest Hospital.

### 2.2 MRI acquisition

A Siemens 3.0-Tesla Trio Tim MRI scanner (Siemens AG, Erlangen, Germany) was used to acquire structural images with a 12-channel phase-array head coil. The subjects were placed in a supine position during image acquisition. The head of each subject was fixed with sponge pads to reduce head movement, and the subjects were asked to keep the head as still as possible during the scan. The following magnetization-prepared rapid gradient echo (MPRAGE) acquisition parameters were used to obtain the 3-dimensional high-resolution structural images: repetition time (TR) = 1900 ms; echo time (TE) = 2.52 ms; inversion time (TI) = 1100 ms; flip angle = 9°; field of view (FOV) = 256 × 256 mm; slice thickness = 1 mm; number of slices = 176; and voxel size = 1 mm × 1 mm × 1 mm.

### 2.3 MRI analysis

Before further analysis of the brain images, we first confirmed that all the images were not influenced by head movement. Structural images were subjected to volume segmentation and cortical surface reconstruction using FreeSurfer software (version 5.3.0, Massachusetts General Hospital, Boston, MA, U.S., http://surfer.nmr.mgh.harvard.edu). The postprocessing procedures have been described in detail in previous studies (Fischl and Dale, 2000; Dale et al., 1999), and were performed separately on each cerebral hemisphere. After the automated imaging processing by the FreeSurfer software, the output of each subject was visually inspected. Whether the surfaces followed gray matter borders and the subcortical segmentation followed intensity boundaries were checked, and if not, all errors were corrected manually for the proper segmentation. Cortical thickness was defined as the shortest straight-line distance between the pial surface and the gray-white matter boundary. The volumes of subcortical regions, including the thalamus, caudate, putamen, pallidum, hippocampus, amygdala, and nucleus accumbens were extracted from the reconstructions.

### 2.4 Statistical analysis

The asymmetry index (AI) was calculated for each cortical and subcortical region to quantify the differences between the left and right hemispheres using the following formula: AI = (left – right) × 100 / (left + right). The AI ranges from −100 (complete right-lateralized asymmetry) to +100 (complete left-lateralized asymmetry). First, the demographic and clinical features and hemispheric asymmetry between the patients with MDD and the NCs were compared. The Mann-Whitney *U* test or independent samples *t*-test was used for parameters that were not normally distributed (e.g., age, education level) or parameters with a normal distribution, (e.g., HAM-D_24_ score, SDS score and SAS score), respectively. The chi-square test was used to assess the differences in gender distribution. Differences in the AI for cortical thickness between the patients with MDD and the NCs were then evaluated using the general linear model (GLM), and a whole-brain statistical threshold correction was performed using the Monte Carlo simulation method (Hagler Jr. et al., 2006) with “sim mc-z” by FreeSurfer. The vertex wise threshold was set at 1.3, and statistical significance was set at a cluster wise corrected *P*-value < .05. To assess significant differences in the AI for subcortical volumes, the Mann-Whitney *U* test was performed using SPSS 22.0 software (IBM Inc., Armonk, New York, USA). Finally, we analyzed the correlations between the AI and clinical features in patients with MDD. The Bonferroni correction was applied to between-group analyses and correlation analyses that involved multiple comparisons.

## 3 Results

### 3.1 Participants' characteristics and hemispheric asymmetry measures

Table 1 shows the demographic information, clinical data and hemispheric asymmetric measures for the MDD and NC groups. The two groups were matched in terms of age, gender, and education (*P* > .05). As expected, patients with MDD had significantly higher HAM-D_24_ scores, SDS scores, and SAS scores than the NCs (*P* < .05). No significant differences in the hemispheric asymmetry of cortical thickness, or subcortical volume were observed between the MDD and NC groups.

**Table 1:** Demographic features and hemispheric asymmetries.

Characteristic	MDD (*n* = 35)	NC (*n* = 35)	Diagnosis effect	*P* value
*Demographic/clinical characteristics*				
Age, years	28.91 ± 1.57	28.11 ± 1.15	*u* = −0.218	0.827
Gender, female: male	22:13	20:15	χ^2^ = 0.238	0.626
Education, years	13.71 ± 0.51	13.91 ± 0.61	*u* = −0.336	0.737
Duration of disorder, years	1.65 ± 0.26	–	–	–
HAM-D_24_ score	30.45 ± 0.68	2.54 ± 0.26	*t* = 38.142	<0.0001
SAS score	56.49 ± 1.94	27.46 ± 0.27	*t* = 14.846	<0.0001
SDS score	61.77 ± 1.57	27.51 ± 0.26	*t* = 21.465	<0.0001

*Hemispheric asymmetries*				
AI of cortical thickness	0.27 ± 0.10	0.11 ± 0.63	*t* = 0.249	0.804
AI of subcortical volume	1.82 ± 0.20	2.19 ± 0.65	*u* = − 0.393	0.694

Abbreviations: MDD = major depressive disorder; NC = normal control; HAM-D_24_ = 24-item Hamilton Depression Scale; SAS = Self-Rating anxiety Scale; SDS = Self-Rating Depression Scale; AI = asymmetry index

Continuous variables are expressed as the mean ± SEM.

Significance *P-*value < .05.

To test differences in age, education and the AI of subcortical volume between MDD patients and NCs, the Mann-Whitney *U* test was applied.

To test differences in HAM-D_24_ scores, SAS scores, SDS scores, and the AI of cortical thickness between MDD patients and NCs, we performed an independent samples *t*-test.

To test the female/male distribution, the chi-square test was used.

### 3.2 Asymmetry

**Fig. 1:**
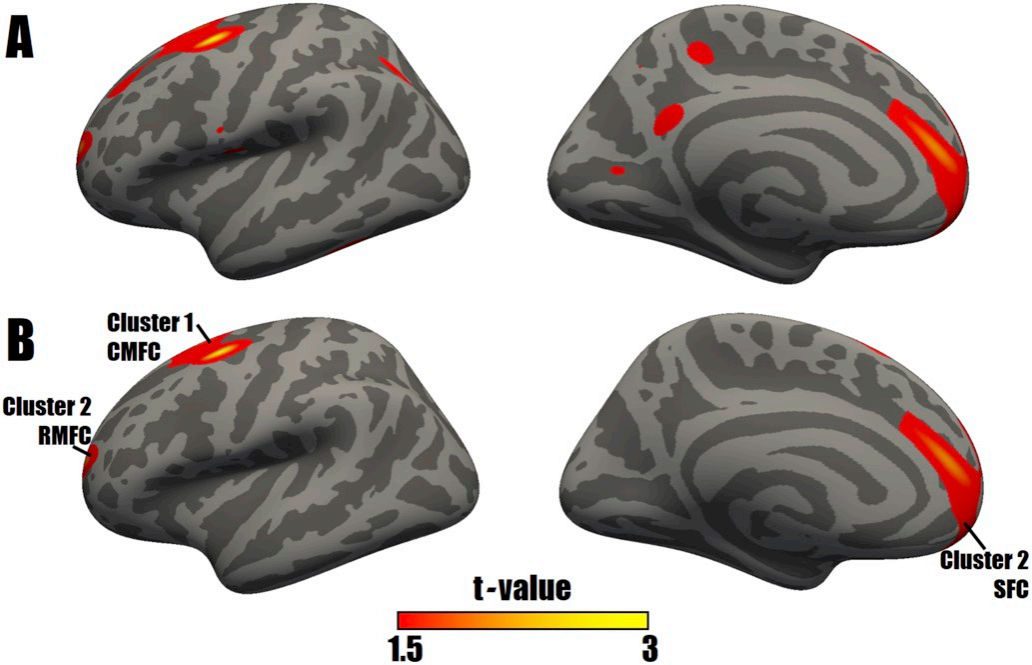
**Uncorrected (A) and corrected (B) surface maps of differences in the AIs for cortical thickness between patients with MDD and NCs.** As determined by a GLM analysis, differences in AIs are presented on left inflated cortical surfaces. Dark gray indicates gyri; light gray indicates sulci. The color bar represents t values ranging from 1.5 to 3. Red to yellow indicates increased AIs in the MDD group compared with those in the NC group; a closer proximity to yellow indicates a greater difference. The numerals refer to the cluster numbers listed in Table 2. The anatomical locations of the peak vertex of clusters 1 and 2 are the caudal middle frontal cortex (CMFC) and superior frontal cortex (SFC), respectively. Otherwise, the lateral surface of cluster 2 involves a portion of rostral middle frontal cortex (RMFC). (For interpretation of the references to color in this figure legend, the reader is referred to the web version of this article.)

According to the GLM analysis, dispersed changes in the AIs of cortical thickness were found in the MDD group (Fig. 1A). After a multiple comparisons correction, only 2 clusters, including the caudal middle frontal cortex, superior frontal cortex and rostral middle frontal cortex, displayed significantly higher AIs for cortical thickness in the MDD group than in the NC group, and no decreases in the cortical thickness AI were detected in the MDD group (Fig. 1B, Table 2).

**Table 2:** Surface-based cluster summary of significant differences in the AIs for cortical thickness between patients with MDD and NCs.

Cluster number	*t*-Value (max)	Size (mm^2^)	MNI coordinates of peak vertex			CWP	CWPLow	CWPHi	Anatomical location of peak vertex
			X	Y	Z				
1	3.025	2813	−30.4	0.8	53.3	0.0012	0.0008	0.0017	Caudal middle frontal cortex
2	2.700	3452	−13	40.7	10.0	0.0001	<0.0001	0.0002	Superior frontal cortex

Abbreviations: CWP, cluster wise *P* value; MNI, Montreal Neurological Institute.

CWPLow and CWPHi: 90% confidence interval for CWP.

**Fig. 2:**
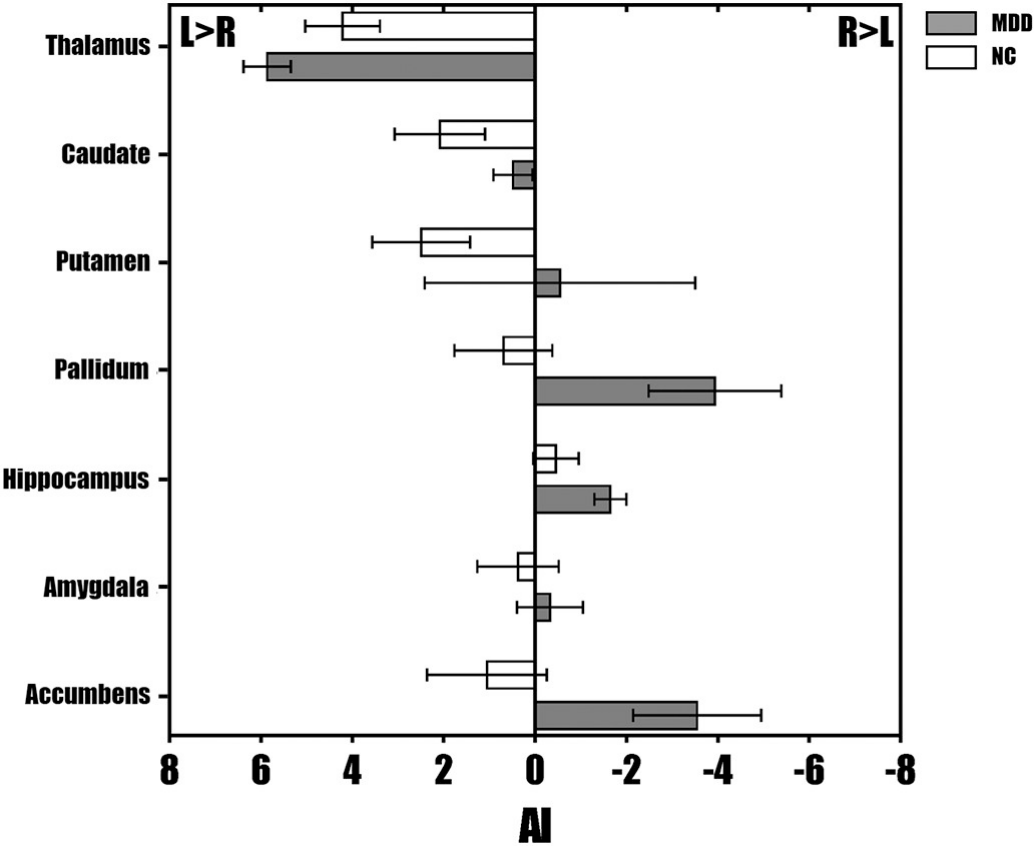
**The average AIs for subcortical volume in patients with MDD and NCs**. The error bars indicate standard errors.

Compared with the NC group, the MDD group showed a greater AI for subcortical volume in the thalamus (*u* = −2.508, *P* = .012) and a lower AI in the pallidum (*u* = −2.132, *P* = .033) and nucleus accumbens (*u* = −2.308, *P* = .021) (Fig. 2). However, after the Bonferroni correction, none of the AIs in subcortical areas showed significant differences between the MDD and NC groups (Bonferroni-corrected *P* > .05).

### 3.3 Correlation analysis

Among the demographic and clinical characteristics of patients with MDD, the only significant positive correlation was detected between the SAS score and the SDS score (*r* = 0.589, *P* < .0001). In patients with MDD, no changes in the AI in brain areas were significantly correlated with symptom severity (using HAM-D_24_, SDS, and SAS scores).

## 4 Discussion

To the best of our knowledge, this study is the first to examine differences in cortical and subcortical asymmetry between treatment-naïve patients with MDD and healthy controls. In the present study, the participants with MDD exhibited more left-lateralized asymmetry in the superior frontal cortex, rostral middle frontal cortex, and caudal middle frontal cortex. The rostral middle frontal cortex and superior frontal cortex are part of the DLPFC, which is critical in coordinating sensory inputs, long-term memory, and aversive memory with executive outputs to enable goal-directed behavior (Barbey et al., 2013). Converging evidence has indicated enhanced attention to and memories of negative emotional stimuli in patients with MDD, potentially due to deficits in the DLPFC (Fales et al., 2008). The DLPFC also plays a key role in the governance, surveillance and integration of cognitive activities. The top-down (cortical-to-limbic) circuitry enables cognitive modulation of emotional processing by the DLPFC. However, cognitive-control and emotional-processing circuitry mostly work in opposition to each other. Completely spontaneous suppression of excessively emotional responses is accompanied by the activation of cognitive-control prefrontal regions such as the DLPFC and a simultaneous reduction in emotional activity in subcortical areas such as the nucleus accumbens (Richard and Berridge, 2013). Therefore, dysfunctional adaptive emotion regulation may be one of the key abnormalities underlying MDD. For instance, in a study of adult subjects by Fales et al. (2008), patients with depression showed an enhanced amygdala response and no DLPFC response while controls showed increased activity in the DLPFC when exposed to affective stimuli, which suggests that depressed patients may have difficulty recruiting prefrontal cortical systems to regulate subcortical regions involved in emotion. Top-down dysfunction was found to be a source of dysregulation in cognitive and emotional processing in depression.

Functional studies have revealed that asymmetric patterns of functional activity in patients with MDD could be hypoactivity and hypometabolism in the left DLPFC and hyperactivity and hypermetabolism in the right DLPFC (Grimm et al., 2008; Phillips et al., 2003). While positive emotion is associated with activation of the DLPFC, particularly in the left hemisphere (Herrington et al., 2005). Neuroimaging studies associated the left DLPFC specifically with approach-related emotions and the right DLPFC with withdrawal-related emotions (Grimm et al., 2008; Balconi and Ferrari, 2012), which is consistent with the valence hypothesis (Wittling and Roschmann, 1993; Davidson and Irwin, 1999). Therefore, alterations in functional asymmetry could be another key role for the physiopathology of MDD. However, our results of the structural asymmetric patterns seem to oppose the functional asymmetric changes in patients with MDD. A greater AI represents a relatively lower cortical thickness in the right than the left side, or a relatively greater cortical thickness in the left than the right side. Therefore, the left-lateralized prefrontal cortex in patients with MDD in our study may reflect that patients with MDD had a relatively thinner right prefrontal cortex, a relatively thicker left prefrontal cortex, or both a thinner right and a thicker left prefrontal cortex than the NCs. Actually, greater activity could occur in a thinner cortex (Anurova et al., 2015), just as lower activity could occur in a thicker cortex (Haier et al., 2009). Alterations in cortical thickness and neuron activity can be inconsistent because cortical thickness may have a greater tendency to be the result of a relatively long-term, chronic accumulation of minor changes, while neuron activity may have a greater tendency to represent an instant, acute functional statement. Several multiple parameter studies have attempted to determine the associations between structural and functional changes in MDD. Although Fonseka et al. (2016) did not prove direct relationships between cortical disturbances and emotional processing, they found that MDD patients with thickening in the left prefrontal cortex showed deficits in emotional performance. Meanwhile, lower cortical thickness in the right prefrontal cortex has been linked to abnormal resting-state functional connectivity of the default mode network (DMN) in patients with MDD (van Tol et al., 2014).

To the best of our knowledge, only two previous structural studies reported greater right-lateralized volumetric asymmetry in patients with MDD than in controls (Kumar et al., 2000; Liu et al., 2016), which seemed to contradict our results. Heterogeneities in patient samples, disease severity, illness duration, medication status, and morphological techniques might contribute to such inconsistencies. However, cortical volume is composed of the cortical thickness and cortical surface area, which are produced by distinct genetic mechanisms, and cortical volume changes are largely driven by gyrification and cortical surface area rather than cortical thickness (Hogstrom et al., 2013). Therefore, a cortical region with rightward asymmetry for cortical volume could have leftward asymmetry for cortical thickness. Otherwise, depressed patients can also show increased activity in the left DLPFC during the Stroop interference task (Killgore et al., 2007), suggesting that compensatory responsiveness mechanisms were recruited to sustain roughly equivalent levels of performance. Similarly, increases in cortical thickness may be one of the compensatory strategies. Therefore, the leftward asymmetry for cortical thickness in the prefrontal cortex of patients with MDD observed in our study represents a course of a chronic pathological trajectory, comparable rightward asymmetry for cortical volume, or a compensatory recruitment mechanism, which requires further exploration.

The bottom-up (limbic-to-cortical) circuitry may impact cognitive systems by sending upward signals to cortical structures. Although the comparison results of the nucleus accumbens, pallidum and thalamus did not survive the Bonferroni correction, which may be overly conservative and result in type II errors (Perneger, 1998; Righart et al., 2013), these subcortical areas also showed a nearly significant trend of the alterations in volumetric asymmetry. They are all involved in the pathway of positive affect processes to affective cognitive systems to create conscious feelings of pleasure: neurons in the nucleus accumbens projects to the pallidum, which in turn projects to the thalamus, which ultimately projects to the prefrontal areas (Berridge, 2003). The nucleus accumbens is an important area that controls locomotion, motivation and the processing of reward and pleasure (Nauczyciel et al., 2013). A previous fMRI study identified a deficit in the ability to maintain nucleus accumbens engagement in patients with MDD during a positive emotion regulation task, indicating that insufficient positive emotion regulation may lead to abnormalities in reward processing and reductions in positive affect (Heller et al., 2009). The authors also found decreased maintenance of functional connectivity between the prefrontal cortex and nucleus accumbens, suggesting impaired upregulation and downregulation of positive emotion in patients with depression. The pallidum is especially likely to execute a hedonic transformation and participates in behavioral motor control, reward, motivation and affective processing (Li et al., 2017). In addition, a reduced ability to gauge the causal consequences of actions among youths with depression has been found to be associated with volumetric reductions in the right pallidum (Griffiths et al., 2015). Both the nucleus accumbens and pallidum are involved in hedonic circuits and are correlated with anhedonia (Wacker et al., 2009; Chung and Barch, 2015), a central and specific feature of depression. Anhedonia is characterized by a lack of reward-motivated behavior and is associated with a decreased experience of pleasure or interest in previously gratifying activities (Pizzagalli, 2014). By targeting anhedonia, treatment outcomes of depression are likely to be improved (Dunn, 2012). The thalamus has not been evaluated in as much detail in functional and morphometric studies of patients with MDD. However, the thalamus is involved in almost all interactions among cortical, subcortical, and brainstem nuclei, and in various functions of the thalamus, such as motor, autonomic, and alerting processes, which are asymmetrical in normal subjects (Ojemann, 1977). Decreased activity in the medio-dorsal thalamus can disrupt prefrontal-dependent cognitive behaviors (Parnaudeau et al., 2013). It is possible that only parts of the thalamus have roles in the experience of emotion, which warrants further investigation.

The different pathologic stages of brain asymmetry could be the cause of some of the depressive symptoms, however, in the present study, the asymmetry in cortical thickness and subcortical volume did not exhibit significant changes as depressive severity increased in patients with MDD, possibly due to the constantly dynamic features of episode severity. Cerebral asymmetry changes could be the result of relatively long-term accumulation and evolution, whereas depressive severity could show various levels in the disease course. A longitudinal observational study (Kovacs et al., 2016) of MDD from childhood to young adulthood demonstrated that none of the traditional demographic factors, clinical variables or treatment exposure variables are reliable predictors of the course of patients with MDD, suggesting that traditional predictors cannot be sufficiently statistically controlled for because they change with over time and across condition. Therefore, since cerebral asymmetry was independent of depressive symptom severity, it may be an appropriate measurement for differentiating patients with MDD from controls.

The present study conclusively showed that interhemispheric structural imbalances in patients with MDD are present in the cortical-striatal-pallidal-thalamic circuit, which regulates positive emotional processes. The clinical treatment of depression has previously focused on diminishing or downregulating negative emotional experiences, and improving or upregulating positive emotional experiences and cognition have only recently been seriously considered (Dunn, 2012). Our work may contribute to the observed development of depression and the clinical diagnosis and treatment of depression. At the genetic level, asymmetry in the expression of gene-stress susceptibility factors such as brain-derived neurotrophic factor (BDNF) may contribute to lateralization in vulnerability to MDD (Farhang et al., 2014; Gatt et al., 2010). Combining structural asymmetry with risk genotypes to predict the occurrence and development of depression could be beneficial in future studies.

Some limitations of this study should be noted. First, our sample size was relatively small, therefore, any asymmetric abnormalities that we identified must be interpreted with caution. A larger number of patients might be useful in obtaining more convincing and accurate results. Second, this study was a cross-sectional investigation, and potential variations in the duration of illness should be studied longitudinally in future studies. Finally, patients with comorbid anxiety were not excluded, which may have affected our results and should be considered.

## 5 Conclusions

In the present study, we utilized an automated surface-based approach and showed that altered morphological interhemispheric imbalances in patients with MDD are mainly located in the cortical-striatal-pallidal-thalamic circuit. These alterations were independent of depressive symptom severity, suggesting that cerebral asymmetry could be an appropriate indicator of morphological variations in mental disease. These findings may provide potential targets for objective diagnosis and therapeutic monitoring for the depression.

## Funding sources

This study was funded by the National Nature Science Foundation of China (grant No. 81171283), the National Key Research and Development Plan of China (grant No. 2016YFC0107101) and the Innovative Talents Project of Southwest Hospital (grant No. SWH2015QN12).

## Conflicts of interest

Nothing to declare.
